# Uncovering Hyperhomocysteinemia: Global Risk Patterns and Molecular Disruption in Brain and Vascular Health

**DOI:** 10.1111/jnc.70327

**Published:** 2025-12-22

**Authors:** Osmar Vieira Ramires Júnior, Gustavo Ricardo Krupp Prauchner, Alessandra Schmitt Rieder, Ana Karla Oliveira Leite, Clarissa Penha Farias, Angela T. S. Wyse

**Affiliations:** ^1^ Programa de Pós‐Graduação em Biociências Universidade Federal de Ciências da Saúde de Porto Alegre (UFCSPA) Porto Alegre Rio Grande do Sul Brazil; ^2^ Programa de Pós‐Graduação em Ciências Biológicas: Bioquímica, Instituto de Ciências Básicas da Saúde (ICBS) Universidade Federal do Rio Grande do Sul (UFRGS) Porto Alegre Rio Grande do Sul Brazil; ^3^ Laboratório de Neuroproteção e Doenças Neurometabólicas, Departamento de Bioquímica ICBS, UFRGS Porto Alegre Rio Grande do Sul Brazil; ^4^ Laboratório de Cognição e Neurobiologia da Memória, Instituto Do Cérebro Pontifícia Universidade Católica do Rio Grande do Sul (PUCRS) Porto Alegre Rio Grande do Sul Brazil

**Keywords:** cognitive dysfunction, endothelial impairment, epigenetic regulation, homocysteine metabolism, neuroinflammation, oxidative stress

## Abstract

Hyperhomocysteinemia (HHcy), a condition characterized by elevated plasma levels of the sulfur‐containing amino acid homocysteine, has emerged as a multifactorial and systemic risk factor with profound effects on neural and vascular integrity. This review integrates recent findings from epidemiological studies, clinical data, and mechanistic research to provide a comprehensive overview of HHcy's contribution to neurovascular dysfunction. We examine how nutritional deficits, aging, genetic polymorphisms—such as in the methylenetetrahydrofolate reductase (MTHFR) and cystathionine beta‐synthase (CBS) genes—pharmacological agents, and comorbid conditions shape homocysteine homeostasis and susceptibility to pathology. Emphasis is placed on molecular pathways, including oxidative and nitrative stress, inflammasome activation, autophagy, and epigenetic modulation, which link HHcy to cognitive decline, memory impairment, endothelial dysfunction, and increased disease burden in neurodegenerative disorders. By consolidating multidisciplinary evidence, we position HHcy as a pivotal but under‐recognized target for intervention in neurochemical and vascular health.

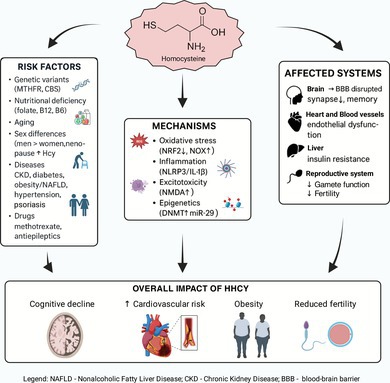

## Graphical abstract

Elevated homocysteine results from genetic variants, impaired one‐carbon metabolism, nutritional deficiencies, and metabolic disorders. Hyperhomocysteinemia drives oxidative stress, inflammation, excitotoxicity, and epigenetic disruption, affecting multiple organs and systems. Clinical consequences include dysfunction of the blood–brain barrier (BBB), increased risk of chronic kidney disease (CKD), and contribution to non‐alcoholic fatty liver disease (NAFLD) progression, as well as cardiovascular, metabolic, and reproductive alterations. Psoriasis is included among the inflammatory conditions associated with elevated homocysteine.

**Abbreviations**
AAA: abdominal aortic aneurysm
ad
: alzheimer's disease
AEDs: antiepileptic drugs
Akt: AKT kinase
AMPA: α‐amino‐3‐hydroxy‐5‐methyl‐4‐isoxazolepropionic acid receptor
AMPK: AMP‐activated protein kinase
AT1: angiotensin II Receptor Type 1
Bax: Bcl‐2‐Associated X Protein
BBB: blood brain barrier
Bcl‐2: B‐cell Lymphoma 2 Protein
BDNF: brain‐derived neurotrophic factor
BMI: body mass index
CAT: catalase
CBS: cystathionine beta‐synthase
CNS: Central Nervous System
CREB: Cyclic AMP response element binding protein
DNA: deoxyribonucleic acid
DNMT: DNA (Cytosine‐5)‐Methyltransferase
eNOS: endothelial nitric oxide synthase
ERKs: extracellular signal‐regulated kinases
GAPDH: Glyceraldehyde‐3‐Phosphate Dehydrogenase
GFR: glomerular filtration rate
GPx: glutathione peroxidase
H_2_S: hydrogen sulfide
H3K4me1: Histone H3 Lysine 4 monomethylation
Hcy: homocysteine
HHcy: hyperhomocysteinemia
HIF: hypoxia inducible factor
HMD: high‐methionine diet
HO‐1: heme oxygenase‐1
HSF1: heat shock transcription factor 1
hTERT: human telomerase reverse transcriptase
IL‐1β: interleukin‐1 beta
KDM1A: lysine‐specific histone demethylase 1A
LINE1: long interspersed nuclear element‐1
LTL: leukocyte telomere length
MAPK: mitogen‐activated protein kinase
MCI: mild cognitive impairment
MDM2: double minute 2 homolog
MIF: macrophage migration inhibitory factor
Mk‐801: dizocilpine maleate
MMP‐9: matrix metalloproteinase 9
MTHFR: methylenetetrahydrofolate reductase
mTOR: mechanistic target of rapamycin
NAFLD: non‐alcoholic fatty liver disease
NFκB: nuclear factor kappa B
NLRP3: NOD‐like Receptor Family, Pyrin Domain Containing 3
NMDAR: N‐Methyl‐D‐Aspartate Receptor
NO: nitric oxide
NOX: NADPH oxidase
NRF2: factor 2 related to NF‐E2
PGC1‐α: peroxisome proliferator‐activated receptor gamma coactivator 1‐alpha
PI3K: phosphoinositide 3‐Kinase
PKC: protein kinase C
PPARγ: peroxisome proliferator‐activated receptor gamma
RDW: red cell distribution range
RNA: ribonucleic acid
ROS: reactive oxygen species
SAP‐97: synapse‐associated protein 97
SAT2: satellite sequence 2
SIAH1: siah E3 ubiquitin‐protein ligase
SOD: superoxide dismutase
T2D: type 2 diabetes mellitus
TAU: microtubule‐associated protein tau
Th1 and Th17: type 1 and type 17 helper T lymphocytes
TrkB: tropomyosin receptor kinase B
TXNIP: thioredoxin interacting protein
VEGF: vascular endothelial growth factor
WMH: white matter hyperintensity

## 1 Introduction

Homocysteine (Hcy), a sulfur‐containing non‐proteinogenic amino acid derived from methionine metabolism, has garnered significant attention as a multifaceted biomarker and pathogenic mediator across diverse clinical conditions. Under physiological circumstances, Hcy homeostasis is maintained via remethylation and transsulfuration pathways, which are dependent on cofactors such as folate, vitamin B6, and B12 (Selhub 1999; Stanger et al. 2009). Disruption of these pathways leads to hyperhomocysteinemia (HHcy), a condition historically linked to cardiovascular disease but now recognized for its broader implications in neurodegeneration, thrombosis, osteoporosis, renal dysfunction, and metabolic disorders (Humphrey et al. 2008; Kim et al. 2018; Bernardi et al. 2024; Li et al. 2025; Alkaissi and McFarlane 2023).

While early studies established HHcy as an independent risk factor for atherosclerosis and stroke (Wald 2002; Homocysteine Studies Collaboration 2002), recent evidence suggests a far more complex and systemic role. Elevated Hcy levels have been implicated in cognitive decline and dementia through mechanisms involving endothelial dysfunction, oxidative stress, and synaptic dysregulation (Ho et al. 2011; Smith and Refsum 2016). Recent studies highlight that HHcy exacerbates vascular injury by impairing antioxidant defenses governed by the Factor 2 related to NF‐E2 (NRF2) pathway and enhancing oxidative stress through NADPH oxidase (NOX) activation (Fan et al. 2019; Wu et al. 2015; Kaplan et al. 2020). Moreover, Hcy interacts with immune‐inflammatory pathways, promoting activation of the NOD‐like receptor family, pyrin domain containing 3 (NLRP3) inflammasome and exacerbating metabolic and vascular injury (Xiang et al. 2023). Emerging research also highlights epigenetic dysregulation, with Hcy acting as a methyl group sink that perturbs deoxyribonucleic acid (DNA) and histone methylation patterns, linking metabolic imbalance to gene expression changes in both vascular and neural tissues (Perła‐Kaján and Jakubowski 2019).

The epidemiology of HHcy is similarly multifactorial and geographically heterogeneous. Prevalence rates vary globally, influenced by dietary folate intake, genetic polymorphisms in enzymes such as methylenetetrahydrofolate reductase (MTHFR), and demographic factors including age, sex, and socioeconomic status (Moat et al. 2004; Clarke et al. 1991, 2010). Notably, the MTHFR C677T variant, prevalent in East Asian and certain European populations, is associated with impaired Hcy metabolism and heightened disease risk (Klerk et al. 2002; Liew and Gupta 2015). Pharmacological agents, including antiepileptics and methotrexate, further modulate Hcy levels, compounding risk in susceptible individuals (Refsum et al. 2004; Linnebank et al. 2011).

Despite these advancements, current literature often remains fragmented—addressing HHcy's vascular, neurocognitive, or inflammatory effects in isolation—without integrating its epidemiological patterns, risk factors, and mechanistic pathways into a unified framework. This gap hampers our ability to develop targeted interventions and public health strategies.

In this review, we aim to provide a comprehensive synthesis of HHcy by critically examining its global epidemiology, multifactorial risk profile, and the intricate signaling networks that link elevated Hcy levels to disease pathogenesis. We integrate recent advances in the understanding of dietary influences, aging, hormonal regulation, genetic susceptibility, pharmacological modulation, and comorbid diseases, while mapping the molecular pathways spanning oxidative stress, inflammation, apoptosis, and epigenetic remodeling. By bridging these domains, we offer a cohesive perspective on HHcy as a central pathological axis, with implications for preventive cardiology, neurology, metabolism, and precision medicine.

## 2 HHcy in a Global Perspective: From Epidemiology to Clinical Implications

### 2.1 Prevalence and Multifactorial Determinants of HHcy

HHcy constitutes an independent risk factor for cardiovascular, neurodegenerative, and thromboembolic events. Epidemiological studies reveal heterogeneous prevalences depending on population context: among Afghan adolescent refugees (15–18 years; cross‐sectional study), 25% presented HHcy, with mean Hcy levels of 14.1 μmol/L in boys and 11.8 μmol/L in girls, associated with vitamin B12 and folate deficiencies; conversely, a 2015 national Ethiopian survey (18–65 years) reported a 38% prevalence, higher in men (49%) than in women (30%), correlating with advancing age, hypertension, and low fruit and vegetable intake (Khan et al. 2022).

Nutritional interventions have proven effective: in older adults aged 50–80 years, combined folate, B6, and B12 supplementation reduced Hcy and improved cognitive function (Chen et al. 2025); in those over 65 years, a multivitamin supplement decreased HHcy regardless of baseline B‐vitamin status (McKay et al. 2000); in vegan adults aged 20–40 years, creatine supplementation reversed Hcy elevations and restored microvascular function (van Bavel et al. 2019).

Genetic and environmental factors further modulate these findings: in hypertensive patients aged 40–70 years, folic acid supplementation was individualized based on the MTHFR C677T genotype, resulting in significant reductions in Hcy and improvements in endothelial function (Ma et al. 2024). In a cohort of adults aged 18–60 chronically exposed to arsenic, plasma Hcy concentrations were significantly elevated compared to unexposed controls, accompanied by disruptions in one‐carbon metabolism and sex‐specific changes in DNA methylation patterns, demonstrating that arsenic exposure directly contributes to HHcy and its epigenetic consequences (Long et al. 2022). The MEGA prospective cohort (30–70 years) found no significant association between HHcy and recurrent venous thrombosis, suggesting that genetic and environmental factors complexly influence thrombotic risk (Hensen et al. 2019). In renal transplant recipients (18–70 years), B‐vitamin therapy reduced Hcy and improved cognitive outcomes in a randomized trial (Scott et al. 2017).

However, methodological heterogeneity—variation in HHcy thresholds (12–20 μmol/L vs. ≥ 15 μmol/L), differences in assay methods (HPLC vs. immunoassay), and diverse study designs (cross‐sectional surveys, cohorts, randomized trials)—hinders direct comparisons and may introduce bias. Pathophysiologically, elevated Hcy promotes endothelial dysfunction via oxidative stress and peroxynitrite formation, DNA hypomethylation, and activation of prothrombotic pathways, explaining its association with atherogenesis, neurodegeneration, and thromboembolic events. Common limitations include small sample sizes in vulnerable subgroups, inadequate control of confounders (diet, medications, comorbidities), and the predominance of cross‐sectional studies limiting causal inference.

To advance the field, standardized surveys should be conducted in underrepresented regions (Latin America, Oceania) with consensus on Hcy thresholds and analytical methods, alongside prospective cohorts to assess HHcy impact on cardiovascular and neurodegenerative morbidity and mortality. Multicenter randomized trials of supplementation (B‐vitamins, creatine) in vulnerable and genetically predisposed populations, together with the incorporation of epigenetic biomarkers, will be crucial to inform globally relevant prevention and treatment strategies.

### 2.2 Clinical Associations and Pathophysiological Mechanisms: The Impact of Hcy on Neurological, Vascular, Infectious, and Reproductive Disorders

Elevated Hcy levels have been increasingly implicated in a wide spectrum of diseases, with mounting evidence highlighting their role in the pathophysiology of neurological, vascular, and infectious conditions. Over the past decade, investigators have sought to unravel the epidemiological complexities of plasma Hcy and its role in neurodegenerative diseases, cardiovascular events, and, more recently, COVID‐19–related complications. This review summarizes studies from multiple regions, populations, and methodologies, underscoring Hcy's value as a biomarker across clinical contexts and the persistent need for rigorous research.

Population‐specific etiology reflects genetic, environmental, and social determinants. In Cuba, early surveys (circa 2000) identified widespread B‐vitamin deficiencies among elders, likely driven by irregular diets and socioeconomic constraints (Lanyau‐Domínguez et al. 2020; Lanyau Domínguez et al. 2006). A cohort study in Havana with 424 individuals aged 65 and older (43 with Alzheimer's disease [AD], 131 with mild cognitive impairment [MCI], and 250 cognitively normal) found that those with AD and MCI had significantly lower levels of vitamins B12, C, and A, and higher Hcy levels compared to the control group. Additionally, the prevalence of HHcy was higher among individuals with cognitive impairment (2020b). Similarly, a South Korean study in Songpa (mean age 74.8 ± 7.2 years; *n* = 321: 100 AD, 100 MCI, 121 controls) linked plasma folate < 3.0 ng/mL, low B12, and elevated Hcy to worse cognitive scores. HHcy and folate deficiency were particularly pronounced in AD participants, reinforcing micronutrient status as a contributor to late‐life cognitive health (Kim et al. 2013).

In Beijing, 92 MCI patients with HHcy received combined folate and B12 supplementation over 24 weeks (January 2015–December 2018), resulting in marked Hcy reductions and cognitive improvement at week 24 (Jiang et al. 2020). However, meta‐analyses of 16 randomized controlled trials (*n* = 6276) found no robust benefit of B12 alone—or B12 plus folic acid ± vitamin B6—on cognitive domains, suggesting that vitamin therapy may not universally translate into cognitive gains (Markun et al. 2021; Piazza et al. 2012; Song et al. 2010).

Genetic factors further modulate Hcy metabolism. The MTHFR C677T polymorphism predicts mild HHcy: homozygotes exhibit higher Hcy than heterozygotes or wild‐type carriers (Levin and Varga 2016; Kang et al. 1991). A Palwal, India, cross‐sectional study (*n* ≈785; age 30–70, predominantly vegetarian) associated HHcy, B12/folate deficiencies, and MTHFR C677T variants with cognitive impairment: 34.3% had MCI, 28.7% moderate, 0.2% severe. HHcy doubled the risk of moderate/severe impairment, while CT heterozygosity and the T allele conferred relative protection (Kaur et al. 2018; Frosst et al. 1995). In elderly Caucasians with MCI (*n* = 359), elevated Hcy and MTHFR 677 T carriage correlated with regional brain atrophy on MRI, implicating gene–Hcy interactions in neurodegeneration (Rajagopalan et al. 2012; Rajagopalan et al. 2011). Beyond MTHFR, transcobalamin II (TCN2) polymorphisms affect B12 transport and Hcy levels. In Chinese ulcerative colitis patients (*n* = 527) versus controls (*n* = 574), TCN2 rs1801198—but not rs9606756—was linked to HHcy, heightened intestinal inflammation, and lower B12/folate status; similar findings emerged in Canada and Greece, though a Turkish cohort found an inverse Hcy–folate correlation, highlighting ethnic and methodological variability (Akbulut 2010; Zezos 2005; Peyrin‐Biroulet et al. 2007; Zheng et al. 2017; Vagianos and Bernstein 2012; Peyrin‐Biroulet et al. 2007; Oberley and Yang 2013; Zheng et al. 2017).

HHcy is a recognized prothrombotic state (Khaliq et al. 2023). In livedoid vasculopathy (*n* = 42), 62% had mild HHcy alongside frequent MTHFR and PAI‐1 variants, with high rates of hypertension (99%) and diabetes (44%) (Marsch et al. 2019; Lussana et al. 2013; Marsch et al. 2019). A thrombosis registry (*n* = 19 678) reported 0.2% with severe HHcy; mean age at diagnosis was 47 years, venous events predominated, and 42% experienced recurrence, underscoring Hcy's prognostic utility in thrombotic risk stratification (Lussana et al. 2013).

Stroke research in Beijing further illustrates Hcy's complexity. In 587 elderly lacunar stroke patients (60–95 years), HHcy independently predicted periventricular—but not deep—white matter hyperintensity (WMH) severity (Wang et al. 2015; He et al. 2014). Among 327 lacunar infarct cases with type 2 diabetes (T2D) (34–91 years), advanced age and recurrent stroke, rather than Hcy alone, drove both periventricular and deep WMH severity (Yu et al. 2018; Yu et al. 2020). Such discrepancies emphasize the need to account for comorbidities like diabetes in assessing Hcy's cerebrovascular impact. Additional vascular outcome studies reveal inconsistent Hcy associations. An acute ischemic stroke cohort found no in‐hospital mortality link but worse functional status at discharge among mildly HHcy patients (Forti et al. 2016; Ma et al. 2014; Forti et al. 2016; Arauz et al. 2014); another high‐risk cohort showed no Hcy–cognitive decline correlation but tied deficits to education and vascular history (Ma et al. 2014). A Mexican post‐stroke sample reported 24% HHcy alongside other modifiable factors (hypertension, smoking, hypercholesterolemia, diabetes) in vascular cognitive impairment and dementia (Arauz et al. 2014).

Traditional cardiovascular risk factors—hypertension, diabetes, dyslipidemia, obesity, smoking—interact with Hcy. In 231 hypertensive patients, MTHFR TT genotype and Hcy ≥ 10 μmol/L increased dyslipidemia odds (Liu et al. 2017; Das and Kaul 2009; Liu et al. 2017). A larger carotid atherosclerosis study (*n* = 1257; ≥ 55 years) demonstrated synergistic effects of HHcy and hypertension on early plaque formation and intima‐media thickening (Zhang et al. 2016). Meta‐analysis data estimate that every 3 μmol/L Hcy reduction yields 24% and 16% risk reductions for stroke and ischemic heart disease, respectively (Wald 2002). Folic acid interventions in hypertensive Chinese cohorts (*n* = 351; amlodipine + folate vs. amlodipine alone) achieved superior Hcy and blood pressure control without increased adverse effects (Bao et al. 2023). A clinical trial involving 858 patients with HHcy demonstrated that daily supplementation with 5 mg of folic acid over 3 months led to a mean reduction of 28.05% in plasma homocysteine levels; however, 43.6% of participants failed to reach normalized values (< 15 μmol/L), with treatment efficacy strongly associated with baseline homocysteine levels and patient adherence to the regimen (Tian, Tian, et al. 2017).

Clinical investigations in other contexts include Parkinson's disease: among 30 patients on Levodopa/Carbidopa intestinal gel (mean follow‐up 42.4 months), 19% developed peripheral neuropathy despite B‐vitamin prophylaxis, with no Hcy correlation or treatment discontinuation (Rispoli et al. 2017). In chronic kidney disease, a six‐trial meta‐analysis (*n* = 2452) found no mortality or cardiovascular benefit from Hcy‐lowering regimens (Nigwekar et al. 2016).

Beyond its established neurological and vascular implications, recent studies have underscored Hcy's detrimental effects on reproductive health, particularly via mitochondrial and epigenetic pathways. In women with polycystic ovary syndrome (PCOS), HHcy has been associated with features of metabolic syndrome and reduced reproductive potential, including altered hormonal profiles and increased miscarriage rates. Mechanistically, HHcy contributes to mitochondrial DNA hypermethylation and impairs respiratory chain function in oocytes, leading to mitochondrial dysfunction and compromised oocyte quality (Jia et al. 2016). In hypofertile men, elevated plasma Hcy levels have also been reported, and supplementation with 5‐methyltetrahydrofolate combined with cofactors supporting the one‐carbon cycle has been shown to significantly improve sperm quality and reduce HHcy (Clement et al. 2023). Together, these findings extend the clinical relevance of Hcy to reproductive endocrinology and highlight its role in gametogenesis and fertility outcomes.

Finally, the COVID‐19 pandemic highlighted HHcy's relevance in novel settings. In a case series of 10 pregnant women with polycystic ovary syndrome and COVID‐19, recurrent miscarriages co‐occurred with plasminogen activator inhibitor elevation, MTHFR polymorphisms, and HHcy; personalized antithrombotic/immunomodulatory therapy yielded favorable outcomes (Jerzak and Szafarowska 2022). Two case reports of young men with COVID‐19–triggered ischemic stroke identified homozygous MTHFR C677T, HHcy, and thromboembolic events, illustrating an interplay between viral hypercoagulability and hereditary thrombophilia (Tabatabaee et al. 2022).

After conducting a comprehensive analysis of the complex interactions related to HHcy, it has become evident that understanding these phenomena requires an integrated approach. This approach must take into account clinical and genetic variables that are specific to each population. The next topic will delve deeper into the elements that can modulate the associations between HHcy and various clinical conditions. By identifying and understanding the risk factors, we will be better equipped to design more targeted preventive and therapeutic strategies to mitigate the impacts of mild HHcy on human health.

## 3 Unraveling the Complexity: Risk Factors Influencing Hcy Regulation in Clinical Settings

Recent studies conducted by our group have explored the intricate relationship between mild HHcy, central nervous system (CNS) (Ramires Junior et al. 2022) and cardiovascular system (Prauchner et al. 2024). While we have examined dietary patterns characterized by high protein content (Silveira et al. 2022), the association between low meat intake and different kinds of diets remains poorly understood. Understanding the regulation of Hcy in clinical contexts requires a multifaceted approach that considers genetic predispositions, nutritional status, demographic variables, pharmacological influences, and disease states. These factors interact dynamically, influencing Hcy metabolism and its associated pathophysiological outcomes.

### 3.1 Exploring Dietary Patterns and Hcy Levels

Research suggests that reducing or eliminating the intake of methionine‐rich animal proteins could lead to lower Hcy levels, as evidenced by studies such as (Velez‐Carrasco et al. 2008), which found significantly lower plasma Hcy concentrations in individuals adhering to vegetarian diets compared to omnivores. Consequently, the absence or limited presence of methionine from animal sources in plant‐based diets may contribute to a more favorable Hcy profile (Hirche et al. 2006), potentially mitigating risks associated with CNS‐related conditions such as AD (Lail et al. 2023). Furthermore, emerging studies suggest that incorporating a variety of plant‐based protein sources may enhance overall nutrient intake while improving cardiovascular risk (Satija and Hu 2018).

However, it's crucial to acknowledge that individuals following diets devoid of animal‐derived proteins may also present lower concentrations of vitamin B12, a key cofactor in Hcy metabolism. Deficiency in vitamin B12 could thus contribute to elevated Hcy levels (Niklewicz et al. 2023). Dietary strategies ensuring adequate intake of B complex vitamins, either through fortified foods or supplements, have shown efficacy in lowering Hcy levels in vegetarians and vegans (Olaso‐Gonzalez et al. 2022). Though contradictory data regarding the outcomes of B12 supplementation exist, a comprehensive understanding of the nutritional components in plant‐based diets and their influence on Hcy metabolism is imperative for deeper insights into the regulation of Hcy plasma levels. Recent findings highlight the importance of regular monitoring of B12 status in individuals following strict vegetarian or vegan diets to prevent potential metabolic disturbances (Rizzo et al. 2016).

### 3.2 Age and Gender: Impacts on Hcy Regulation

Besides dietary patterns' influence on Hcy plasma levels as a risk factor, aging and age‐related conditions may enhance the effects of the condition (Ostrakhovitch and Tabibzadeh 2019), as aging relates to lower enzymatic function (Pastoris et al. 2000) and compromised protein transcription/activity (Anisimova et al. 2018). In this regard, as HHcy compromises an important condition detrimental to such parameters (Tyagi et al. 2005; Oudi et al. 2010), and aging represents a contributor to the condition's effects on several tissues and systems, a major element poorly understood can be brought forth, as from the general population, with a prevalence of 5%–7% for the condition, the most affected is the elderly population (Smith and Refsum 2016).

Conditions, like an increased bone fragility and a higher risk of fractures in the elderly (Behera et al. 2017), neurological and neurodegenerative processes (Behera et al. 2017), as well as Age‐Related Macular Degeneration (Huang et al. 2015) are examples of what may culminate from the interplay between the potential risk factor of mild HHcy and aging, and are, despite that, still overlooked during animal model experiments, with most preclinical studies being carried out in young male animal models, which have demonstrated, for example, alterations in at least one of the antioxidant enzymes: superoxide dismutase (SOD), catalase (CAT), or glutathione peroxidase (GPx) in the heart (Longoni et al. 2017), as well as in the damaged endothelium (Wu et al. 2015) and CNS (Ramires Junior et al. 2022). Nonetheless, despite such findings, the influence of age represents a key element to be evaluated and added to the present existing data.

Gender differences are also evident; females, particularly premenopausal women, tend to have lower Hcy levels, partly due to the protective effects of estrogen (Xu et al. 2020). Postmenopausal women experience increased Hcy, aligning with the decline in estrogen levels. According to a cross‐sectional study carried out by Cheng and collaborators, sexual dimorphisms contribute to HHcy being associated with a greater risk in males, with female sex‐specific characteristics being attributed protection to the condition (Cheng et al. 2022). However, it can also be observed that, for every 5 μM increase in Hcy levels, there is a corresponding 40% increase in the risk of isolated diastolic hypertension, a characteristic that is more significant for women compared to men (Kalra 2004).

Pregnancy introduces physiological changes that typically lower Hcy levels, but a history of hypertensive disorders during pregnancy can elevate Hcy, increasing risks for adverse outcomes (Gaiday et al. 2018). Furthermore, an epidemiological study carried out in women aged 60.60 ± 12.46 years highlighted the protective role of estradiol against HHcy (Niu et al. 2022). In this sense, there is still contrasting data on hormonal factors and the estrous cycle influence in mild HHcy.

### 3.3 Pharmacological Influences and Disease States

Medications can significantly alter Hcy metabolism. For example, methotrexate, used in autoimmune diseases and cancer, inhibits dihydrofolate reductase, impairing folate regeneration and elevating Hcy (van Ede et al. 2002; Svardal et al. 1988). Conversely, B‐vitamin supplementation via injections or oral routes can mitigate HHcy, reducing the risk of stroke and cardiovascular events (Chen et al. 2020). Understanding the pharmacological influences on Hcy is vital in both managing existing conditions and preventing associated complications.

Certain medications, such as antiepileptic drugs (AEDs) like phenytoin and valproate, have been shown to disrupt folate metabolism, leading to elevated Hcy levels (Reynolds 2024). These AEDs can interfere with the absorption and utilization of folate, which is crucial for the remethylation of Hcy to methionine. Additionally, the use of some diuretics has been associated with increased Hcy concentrations, potentially heightening the risk of cardiovascular diseases in patients (Morrow and Grimsley 1999). On the other hand, emerging research suggests that statins, commonly prescribed for hyperlipidemia, may have a beneficial effect on Hcy levels by enhancing endothelial function and promoting vascular health (Ridker et al. 2002; Liu et al. 2023). Therefore, a comprehensive understanding of how various pharmacological agents influence Hcy metabolism is essential for optimizing treatment strategies and minimizing the risk of Hcy‐related complications in patients with chronic diseases.

### 3.4 Disease Contributions to Hcy Complexity

Similarly, various diseases contribute to the complexity of Hcy regulation. Conditions such as renal failure, hypothyroidism, and psoriasis are known to elevate Hcy levels. In chronic kidney disease, for instance, impaired renal clearance leads to Hcy accumulation in the bloodstream (Catargi et al. 1999). Additionally, because thyroid hormones enhance renal blood flow and increase the glomerular filtration rate (GFR), hypothyroidism—characterized by reduced GFR—can also result in elevated plasma Hcy levels (Pan et al. 2021).

Inflammatory diseases like psoriasis add another layer to this complexity by highlighting the interplay between Hcy and immune‐inflammatory pathways, particularly involving type 1 and type 17 helper T lymphocytes (Th1 and Th17) cells (Lin et al. 2019). As inflammation progresses, the release of cytokines and other inflammatory mediators can disrupt the activity of enzymes such as matrix metalloproteinases. This disruption may contribute to tissue remodeling, as seen in hidradenitis suppurativa—a chronic, inflammatory skin disease—which, through these mechanisms, represents an additional risk factor (Molnar et al. 2020).

Moreover, diabetes mellitus has also been linked to increased Hcy levels, particularly in individuals with poor glycemic control. Hyperglycemia can induce oxidative stress and endothelial dysfunction, both of which impair Hcy metabolism and clearance (Hu et al. 2019). In T2D, elevated insulin resistance has been associated with reduced expression of key enzymes involved in Hcy remethylation and transsulfuration, thereby promoting its accumulation (Tessari et al. 2005). Similarly, neurodegenerative conditions such as AD have shown correlations with elevated Hcy, likely due to the interplay between oxidative stress, vascular dysfunction, and impaired one‐carbon metabolism (Smith and Refsum 2016). These disease‐specific mechanisms emphasize that elevated Hcy is not only a biomarker but also a potential mediator of pathological processes in diverse clinical contexts.

### 3.5 Genetic Factors and Lifestyle Influence

In this context, genetic mutations help to unravel the biomolecular basis of Hcy dysregulation. One well‐studied example is the MTHFR gene mutation C677T, which is relatively common among white and Hispanic populations—with heterozygosity observed in approximately 20%–40%—and rare among Black individuals (1%–2%). This mutation is associated with a reduction in enzymatic activity to about 65% of normal. When present in both alleles (homozygous), as seen in roughly 8%–20% of individuals from North America, Europe, and Australia, enzymatic function drops further to approximately 30% of normal (Moll and Varga 2015). Another variant, MTHFR A1298C, may also contribute to mild elevations in Hcy levels. In both cases, ethnicity plays a significant role in determining mutation frequency, and combinations of these mutations can co‐occur.

Beyond MTHFR, mutations in the cystathionine β‐synthase (CBS) gene are also relevant. The two most prevalent CBS variants—pyridoxine‐responsive I278T and pyridoxine‐nonresponsive G307S—are primarily associated with severe HHcy, known as homocystinuria (Kraus et al. 1999).

The complex interplay between genetic predisposition, lifestyle factors, and Hcy metabolism further complicates the landscape. Individuals carrying certain genetic variants may be more vulnerable to developing HHcy, highlighting the potential value of personalized medicine approaches for prevention and intervention.

**FIGURE 1:**
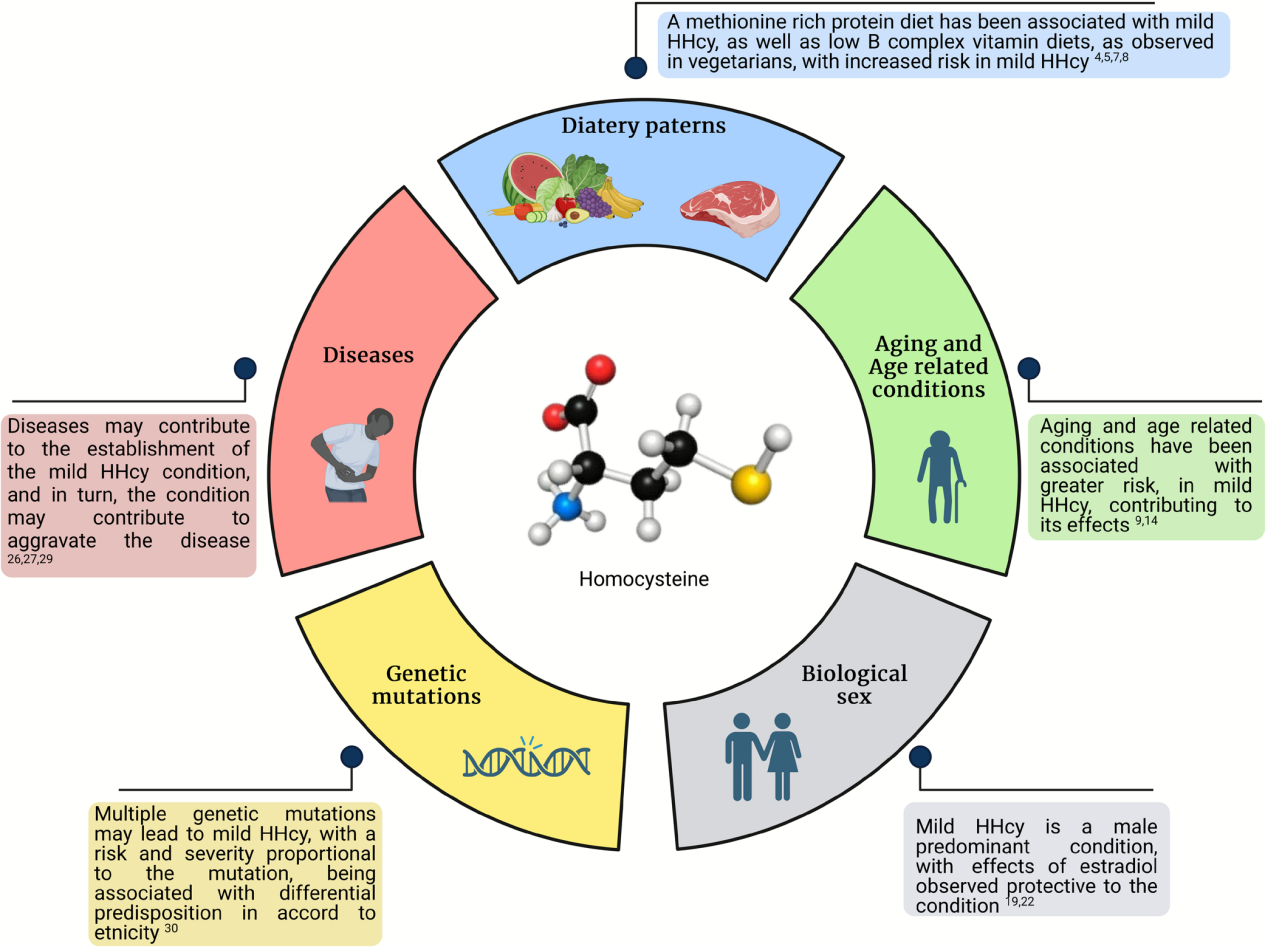
Highlight of the principal risk factors associated with hyperhomocysteinemia (HHcy) in relation to diet, age, sex, genetic predisposition, and the presence of any disease associated with HHcy. The references are displayed with the corresponding risk factor.

However, the interpretation of these risks and their relative contributions is often limited by confounding variables and underreporting. HHcy appears to be more prevalent in males (Cheng et al. 2022), and outcomes may be influenced by factors such as increased body mass index (BMI), smoking habits, and daily fruit intake—all of which are frequently underrepresented or overlooked during data collection (Yang et al. 2021). Unfortunately, data addressing these issues in mild HHcy remain scarce, limiting the ability to draw firm conclusions. Future research aimed at filling these gaps may refine our understanding of risk indicators and their broader clinical implications (Figure 1).

## 4 Elucidating Complex Pathways: Molecular and Behavioral Implications of HHcy in Neurological and Cardiovascular Disorders

HHcy is known to be related to behavioral impairments such as anxiety‐like symptoms (dos Santos et al. 2022). The impairments extend to increased oxidative stress, DNA damage, and atrophy in the hippocampus, as well as compromised mitochondrial function in the amygdala, reduced synapsin 1 protein content, and an altered neuroinflammatory picture (Wyse et al. 2020; Ramires Junior et al. 2022; dos Santos et al. 2019).

Importantly, HHcy alters acetylcholinesterase activity differently depending on brain structure, decreasing in the cerebellum and increasing in the striatum (dos Santos et al. 2021). These cholinergic modifications correlate with delayed motor and cognitive impairments characteristic of HHcy‐related neurodegenerative disorders like Parkinson's and AD (Al‐kuraishy et al. 2023).

The outcomes presented above have been extensively reviewed by many researchers (Kaplan et al. 2020; Guieu et al. 2022). The inflammatory and oxidative effects of this pathology are the main topics described in the literature (Ji and Kaplowitz 2004; Tawfik, Elsherbiny, et al. 2021). However, the epidemiological aspects and risk factors, as well as the pathways underlying HHcy, have been poorly reviewed. Given that gap, here our focus will be on these three key aspects of the disease.

### 4.1 Oxidative and Nitrative Stress Pathways

HHcy has been related to several pathways, including those related to antioxidant, apoptotic, energetic, and metabolic pathways. The NRF2, which regulates the antioxidant response, is reduced in many HHcy models (dos Santos et al. 2019; Navneet, Zhao, et al. 2019; Cao et al. 2021; Li, Cheng, et al. 2018). Recently, studies in NRF2 knockout mice revealed that the inner retinal layer of rats treated with Hcy was thicker and that the visual acuity was impaired. Also, in in vitro studies, the cells lacking NRF2 do not show the same mitochondrial and glycolytic alterations that healthy cells do when exposed to HHcy; these findings suggest that NRF2 is essential for mediating the effects of HHCY (Navneet, Zhao, et al. 2019). Differently, Navneet and colleagues found that elevated Hcy reduced NRF2 levels in Müller cells. The authors suggest that this may reflect the early stages of the Hcy effect (Navneet, Cui, et al. 2019).

Heme oxygenase‐1 (HO‐1) is one of several antioxidant enzymes activated downstream of NRF2 (Dodson et al. 2019). In a hypertension model, HHcy enhanced the effects of the disease, suppressing both NRF2 and HO‐1 in rats' cardiac tissue. This suppression impaired NRF2 translocation, enhancing collagen deposition in the cardiac tissue causing oxidative stress (Cao et al. 2021). This metabolic imbalance can also suppress lipolysis through NRF2‐mediated mechanisms. Similar findings were made by Li and colleagues (2018), who showed an increase in both the expression and protein content of NRF2 in fully differentiated 3 T3‐L1 adipocytes and in animal models exposed to Hcy (Li, Cheng, et al. 2018). Moreover, the same research group showed that Hcy inhibits lipolysis via AMP‐Activated Protein Kinase (AMPK), an important energy sensor (Wang et al. 2011). Summarizing, HHcy compromises antioxidant defenses, promotes oxidative stress, and causes energy imbalance. These effects are also related to metabolic disorders such as diabetes and hypertension (Alkaissi and McFarlane 2023; Esse et al. 2019).

The phosphoinositide 3‐kinase (PI3K)/AKT kinase (Akt)/NRF2/HO‐1 in addition to other pathways is intimately involved in oxidative stress control and HHcy (Zhang, Liu, et al. 2021; Cui et al. 2019; Chu et al. 2021). Hcy was capable of reducing the Akt phosphorylation levels as well as endothelial nitric oxide synthase (eNOS) phosphorylation and nitric oxide (NO) formation. This signaling pathway, including Akt/eNOS/NO, is related to cell survival (Wu et al. 2019). The antioxidant molecule Oxymatrine was capable of restoring the eNOS levels, but not reversing the Akt levels, which can activate other pathways generating Hcy effects (Wu et al. 2019). The Akt activation induces phosphorylation and inactivation of GSK3β. This pathway regulates glucose metabolism in the brain (Feng et al. 2021; Zhang, Lachance, et al. 2021). Ramires and colleagues (Ramires Júnior et al. 2023) showed that Hcy impaired glucose metabolism by dysregulation of the Akt/GSK3β/GLUT1 signaling pathway and promoted an increase in oxidative/nitrative stress in slices treated with Hcy. This work also showed impaired glucose uptake and reduced levels of GLUT1 transporter, as well as that Hcy causes an imbalance in Na+, K+‐ATPase homeostasis and leads to astrogliosis. Longoni et al. (2018) corroborate these findings by showing that HCY induced glial reactivity by activating nuclear factor kappa B (NFκB) and inhibiting HO‐1 in adult rat astrocyte cultures (Ramires Júnior et al. 2023; Longoni et al. 2018). In cardiac tissue, Hcy led to collagen deposition in the coronary arterioles and elevated blood glucose linked to insulin resistance (Huo et al. 2018). The study reported that increased methionine intake resulted in lower NO levels, probably due to enhanced superoxide anion generated in HHcy that reacts with NO to form the peroxynitrite (dos Santos et al. 2019). Recently, HHcy was associated with neuronal damage and loss in the hippocampal CA3 region by downregulating Cyclic AMP Response Element Binding Protein (CREB) phosphorylation. The CREB phosphorylation can be induced by protein kinase C (PKC) and the same group reported that HHcy downregulated the expression level of phosphorylated PKC (Liu, Zhao, et al. 2022; Zhao et al. 2015). Therefore, the mechanism that regulates HHcy in cardiac dysfunctions shares some aspects with neural HHCY effects (Prauchner et al. 2024).

### 4.2 Inflammation Pathways

Most pathways affected by HHcy are associated with cardiovascular dysfunctions, including hypertension, non‐alcoholic fatty liver disease (NAFLD), and neurodegeneration (Ganguly and Alam 2015; Bonetti et al. 2016; Pastore et al. 2014; Liu et al. 2024; Ramires Júnior et al. 2023). Recent studies have shown that NLRP3 inflammasome activation by NF‐κB contributes to NAFLD in HHcy, up‐regulating the double minute 2 homolog (MDM2), which ubiquitinates heat shock transcription factor 1 (HSF1). The HSF1 activated hepatic NLRP3 inflammasome expression in a dose‐ and time‐dependent manner (Xiang et al. 2023). In addition, Hcy upregulates caspase‐2, ASL, IL‐1β, IL‐18 expression, and NOX pathway through NLRP3 activation (Xiang et al. 2023; Liu, Tao, et al. 2022). The activation of NLRP3 was found in the presence of lipid rafts that generate reactive oxygen species (ROS) and recruit caspase‐1 (Abais et al. 2014; Dai et al. 2021). In HHcy cellular models, caspase‐1 activation has been linked to mitochondrial membrane potential collapse, cytochrome‐c release, and increased Bcl‐2‐associated X protein (Bax)/B‐cell lymphoma 2 protein (Bcl‐2) ratio mediated by oxidative stress (Dodson et al. 2019). Researchers evaluated the role of thioredoxin interacting protein (TXNIP) inhibition in NLRP3 inflammasome activation. In podocyte cell cultures, they observed that verapamil (a TXNIP inhibitor) abolished TXNIP recruitment to NLRP3, as well as the increases in caspase‐1 activity and interleukin‐1 beta (IL‐1β) production normally induced by Hcy. In an in vivo model, combined verapamil treatment and TXNIP transfection ameliorated proteinuria, albuminuria, glomerular damage, and podocyte injury caused by HHcy. In addition, the damage associated with Hcy metabolites, such as the SAH toxicity, can be ameliorated with TXNIP transfection in a diabetic model (Abais et al. 2014; Cao et al. 2013; Dai et al. 2021). The NLRP3 inflammasome drives IL‐1β generation (Wang et al. 2017), which in an HHcy animal model was upregulated along with IL‐6, elevated acetylcholinesterase activity, and reduced nitrite levels (Longoni et al. 2018). The HHcy IL‐1β‐induced was related to a sucrose complex SNF5 through lysine‐specific histone demethylase 1A (KDM1A), a lysine histone demethylase that represses histone H3 lysine 4 monomethylation (H3K4me1) and can demethylate the H3K4me1 (Xie et al. 2021). This enzyme can also methylate IL‐1β residues (Xie et al. 2021; Qureshi and Mehler 2010). Moreover, there is extensive discussion about the methylation outcomes related to the imbalance of the methionine‐Hcy cycle. The NLRP3 is also involved in glomerular damage and proteinuria induced by HHcy, both of which were ameliorated by folic acid supplementation (Cao et al. 2013). These findings suggest that HHcy‐related inflammation likely results from NLRP3 activation mediated by ROS formation, involving multiple signaling pathways and proteins.

### 4.3 Hcy and Glutamate

Interestingly, HHcy has also been linked to N‐methyl‐D‐aspartate receptor (NMDAR) activation in the retinal endothelium and cerebral regions of rats (Tawfik, Mohamed, et al. 2021; dos Santos et al. 2022). In retinal endothelial cells, the CBS knockout mice (an HHcy model) exhibited reduced Zonula Occludens and occludin expression, together with reduced retinal albumin, impairing cell permeability. These effects were reversed with Dizocilpine maleate (Mk‐801) administration or in NMDAR knockout mice (Tawfik, Mohamed, et al. 2021). Similarly, Dos Santos and colleagues found that the Mk‐801 pretreatment prevented HHcy‐induced alterations in amygdala and prefrontal cortex slices. Moreover, the HHcy disrupted glutamate uptake, glutamine synthetase activity, Na + K + ATPase function, and impaired cytochrome c oxidase activity, probably via NMDA receptors overactivation (dos Santos et al. 2022). Both studies confirmed that HHcy effects were at some point driven by NMDAR activation.

### 4.4 Apoptotic and Autophagic Implications

In human umbilical vein endothelial cells, Hcy treatment triggered autophagy through macrophage migration inhibitory factor (MIF)/mechanistic target of rapamycin (mTOR) pathway. MIF, a cytokine secreted by T lymphocytes, inhibits mTOR that normally blocks autophagy and apoptosis via PI3K protein and Bcl‐2 and Bax family, inducing apoptosis throughout the mitochondrial pathway (Zhang et al. 2018). In HHCY models with CBS deficiency, decreased Akt activation within the mTOR pathway may impair post‐ischemic recovery, potentially due to inappropriate hypoxia inducible factor (HIF) and peroxisome proliferator‐activated receptor gamma coactivator 1‐alpha (PGC1‐α) signaling (Veeranki et al. 2014; Liu et al. 2024). HIF is a hypoxia‐responsive transcription factor that plays an essential role in the cellular response to low oxygen (McGettrick and O'Neill 2020; Koh et al. 2022), and PGC1‐α overexpression increases anaerobic glycolysis in a PPARβ‐dependent manner. Furthermore, Veeranki et al. (2014) showed that HHcy impaired angiogenesis in muscular fibers via reduced HIF and PGC1‐α. Leptin (a HIF‐activated endothelial proliferative factor) and CD31 glycoprotein (an endothelial marker) were also decreased, which corroborates the findings (Veeranki et al. 2014; Huo et al. 2018). In an animal model of higher methionine intake, the animals exhibited reduced peroxisome proliferator‐activated receptor gamma (PPARγ) expression in coronary arterioles due to poor post‐ischemia recovery and autophagy suppression.

Moreover, glyceraldehyde‐3‐phosphate dehydrogenase (GAPDH), a key glycolytic enzyme, exhibits altered nuclear translocation under HHcy conditions. This protein plays a role in apoptosis induction through p53 activation (Fang et al. 2016; Chu et al. 2025; Adtani et al. 2025). Researchers discovered that SIAH E3 ubiquitin‐protein ligase 1 (SIAH1), which contains a nuclear localization signal, facilitates GAPDH translocation in C6 cells. SIAH1 is implicated in DNA damage response, tumor suppression, and apoptosis regulation (Wu and Yan 2024). The study revealed that Hcy enhances both postulated p53 and total p53 levels, with SIAH1 responsible for p53 phosphorylation and activity modulation. Additionally, Hcy treatment increased caspase‐3 cleavage, an apoptosis marker, and elevated Hcy levels were associated with GAPDH nuclear accumulation, likely mediated by SIAH1 (Itakura et al. 2015; Fang et al. 2016; Tian et al. 2019). These findings suggest that targeting SIAH1 could provide neuroprotection by mitigating cellular injury.

### 4.5 Vascular Effects and the Renin‐Angiotensin‐Aldosterone System

Clinical studies have consistently associated lower extremity deep vein thrombosis and abnormal aortic aneurysm (AAA) with HHCY, including studies regarding genetic MTHFR alterations as well as life style conditions (Liu et al. 2016; Zhang et al. 2023). Yu and collaborators demonstrated that Vascular Endothelial Growth Factor (VEGF) mediates this association (Yu et al. 2019). In HUVEC cells, Hcy impaired cellular migration and viability, effects that were suppressed by VEGF‐inhibitor ribonucleic acid (RNA) transfection. In the AAA model, T. Li and colleagues showed that Hcy binds to both orthosteric and allosteric sites on the angiotensin II type 1 receptor (AT1), blocking receptor activation and potentially exacerbating cardiovascular outcomes in HHcy (Li, Yu, et al. 2018; Lopez et al. 2023). Ang II is the primary mediator of the renin‐angiotensin‐aldosterone system and acts in the AT1 receptor, a G‐protein‐coupled receptor. The study describes that Hcy saturation reduces PKC and extracellular signal‐regulated kinases (ERKs) phosphorylation when compared with AngII saturations. In this view, small molecules that could block AT1 receptors in the binding site of Hcy could ameliorate the HHcy related to AAA, since Hcy binds to the AT1 receptor, a vascular outcome worse (Li, Yu, et al. 2018).

### 4.6 Hcy and Epigenetics: From Animal Models to Clinics

It is known that the enzyme DNA methyltransferases play a role in global DNA methylation and may be related to these alterations through DNA (cytosine‐5)‐methyltransferase (DNMT) 1/2 binding to CBS (Behera et al. 2019; Derouiche et al. 2014; Singh et al. 2022). Hcy has also been associated with endothelial cell dysfunction in mice fed a high‐methionine diet (HMD), due to hypermethylation of the CBS promoter, as well as impaired cell adhesion, angiogenic functions, and blood flow. In HMD blood marrow‐derived endothelial cells, the treatment resulted in higher levels of Hcy and low levels of hydrogen sulfide (H_2_S), a product of the CBS enzyme. The 5′‐Aza, a molecule that promotes CBS demethylation, can restore blood flow and endothelial properties in the HMD animal model (Behera et al. 2019).

Expression analysis revealed that HHcy upregulates miRNA‐29 and DNMT3B, but not DNMT3A. miRNA‐29 acts as a negative epigenetic modulator of genes responsible for encoding tight junction proteins and antioxidant enzymes and Nrf2 protein (Kalani, Kamat, Familtseva, et al. 2014; Heidari‐Kalvani et al. 2025). ROS promotes miRNA29 transcription, which blocks the matrix metalloproteinase 9 (MMP‐9) repressor DNMT3B, consequently increasing its expression and activity, leading to blood‐brain barrier (BBB) permeability. Furthermore, other studies have shown that Hcy causes an imbalance between MMP and its tissue inhibitor, tissue inhibitor of metalloproteinase 1, in diabetic retinopathy. Human retinal endothelial cells and animal models have demonstrated that HHcy exacerbates hyperglycemia‐induced effects and apoptosis linked to MMP‐9 activation, while HHcy regulation can reduce or prevent diabetic retinopathy outcomes (Mohammad and Kowluru 2020). Kalani and collaborators also found that HHcy is capable of reducing CD32, VE‐cadherin, and occludin, which are important markers of BBB stability. The NOX‐4, an oxidative stress marker, was elevated in the HHcy model, while eNOS (an endothelial marker) and Synapse‐Associated Protein 97 (SAP‐97) (a synaptic plastic marker) were reduced. The 5′‐Aza treatment also improved some of these outcomes (Kalani, Kamat, Givvimani, et al. 2014; Kalani et al. 2013; Kalani, Kamat, Familtseva, et al. 2014).

Additionally, H_2_S has been shown to influence HHcy outcomes. The NaHS exerted a protective effect on BBB dysfunction by inhibiting the upregulation of MMP‐9 RNA expression and activity, which were elevated in cerebral regions of HHcy rats, and by reducing cerebral edema in these animals (Kumar and Sandhir 2022). In hypertensive patients, individuals with HHcy exhibited higher blood pressure, and this measure was positively correlated with serum Hcy levels. Regarding lipid parameters, HHcy patients had lower HDL‐cholesterol. The same study, conducted in Wistar rats, with normal and elevated blood pressure, demonstrated that Hcy exacerbates hypertension by increasing blood pressure, whereas folate treatment reduces blood pressure levels. Furthermore, HHcy was correlated with downregulation of H_2_S (Zhao et al. 2021; Shi et al. 2019).

On the other hand, HHcy is associated with lower vitamin B12 and folate levels and contributes to leukocyte telomere length (LTL) shortening, which is linked to hypomethylation of the human telomerase reverse transcriptase (hTERT) gene in essential hypertension. Interestingly, researchers found that the LTL reduction and hTERT alterations were primarily associated with HHcy, followed by age and essential hypertension (Zhang et al. 2014). In the clinical study, the prevalence of HHcy was higher in men than in women, and the associated markers were different between sexes. In women, HHcy was related to a higher BMI and visceral fat, whereas in men, it was associated with lower red cell distribution range (RDW) and HDL levels, as well as a higher LDL/HDL ratio. The mean age of this study was 18 years. Other studies involving individuals older than 40 years demonstrated sex‐specific differences in long interspersed nuclear element‐1 (LINE1) and satellite sequence 2 (SAT2) methylation, with higher prevalence in men with elevated BMI visceral fat, as well as in postmenopausal women, suggesting a hormonal influence on epigenetic regulation. The same study showed that LINE1 and SAT2 methylation levels correlated with higher methionine levels and varied depending on folate levels (Hernandez‐Landero et al. 2022).

**FIGURE 2:**
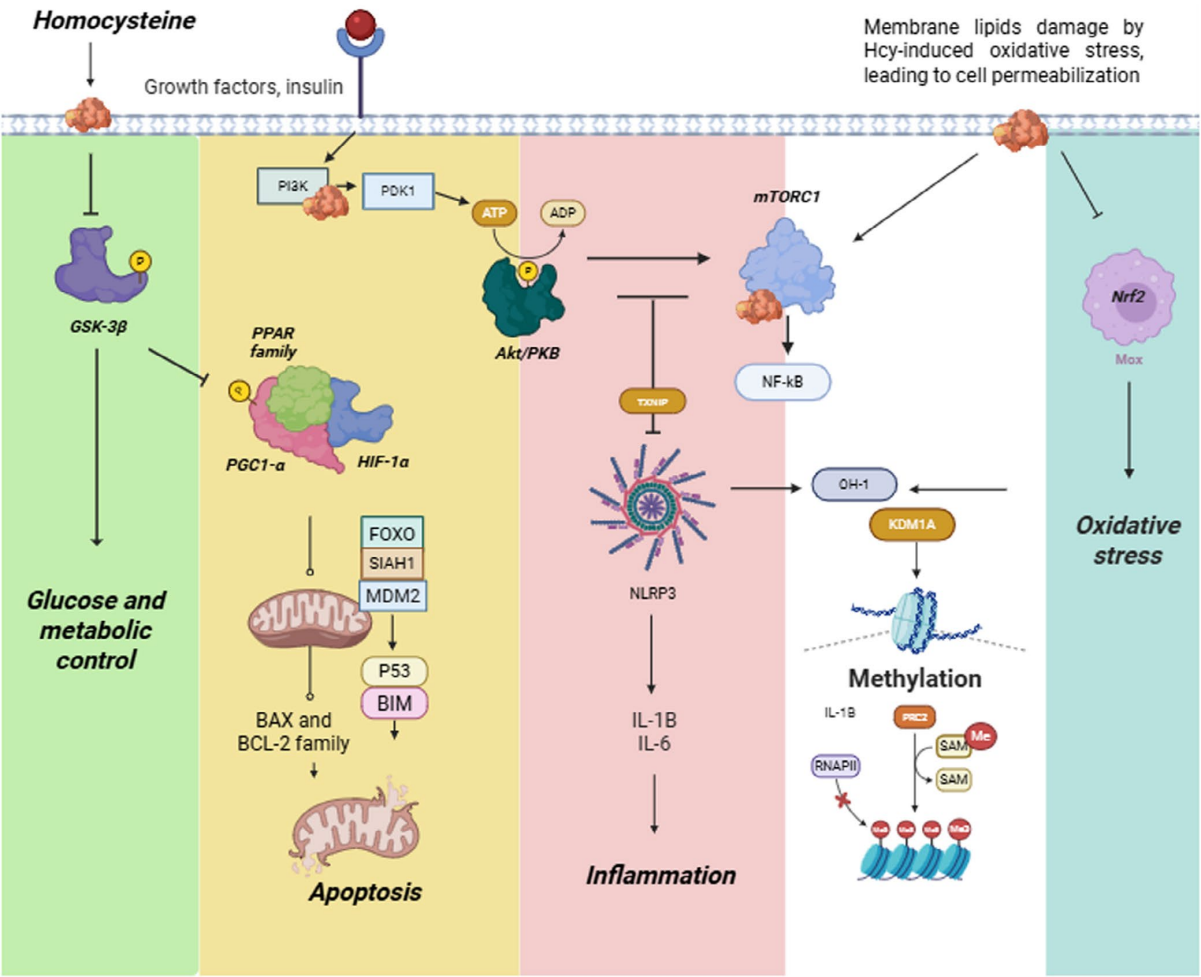
Representative figure of key pathways altered by homocysteine. Each pathway is composed of signaling proteins that have been previously reported to be dysregulated in hyperhomocysteinemia (HHcy). Metabolic dysfunction, oxidative stress, and inflammation occur in conjunction with epigenetic modifications. Together, these disrupted pathways contribute to HHcy‐related pathologies, which are primarily associated with neurodegenerative and cardiovascular diseases.

Visualizing Hcy‐affected proteins and pathways enhances our understanding of HHcy pathophysiology. The interconnected mechanisms described likely synergize to promote HHcy development. When combined with individual risk factors, these pathways may explain the heterogeneous distribution of HHcy. Figure 2 illustrates the interplay between Hcy's effects and the crosstalk among these pathways, offering a comprehensive perspective on HHcy's molecular basis.

While molecular mechanisms such as oxidative and nitrative stress, inflammatory activation, and epigenetic dysregulation have been recognized as major contributors to HHcy–induced neurovascular damage, accumulating evidence suggests that not all toxic effects of homocysteine arise from its free circulating form. Beyond its impact on DNA methylation and gene regulation, a considerable portion of homocysteine's cytotoxicity is mediated by reactive intermediates generated during amino acid misactivation. Among these, homocysteine thiolactone (Hcy‐thiolactone) has emerged as a crucial metabolite that directly links disturbed homocysteine metabolism to aberrant protein modification, misfolding, and cellular stress. The following section delves into the biochemical reactivity of Hcy‐thiolactone, its interaction with antioxidant systems and detoxifying enzymes, and the downstream consequences of N‐homocysteinylation in neural and vascular tissues.

### 4.7 Homocysteine Thiolactone: A Reactive Intermediate in Protein Modification and Neurovascular Toxicity

Homocysteine thiolactone (Hcy‐thiolactone) represents a key molecular link between disrupted sulfur amino acid metabolism and protein damage in HHcy. Formed through the error‐editing activity of methionyl‐tRNA synthetase, Hcy‐thiolactone is a reactive cyclic thioester capable of non‐enzymatically acylating the ε‐amino group of lysine residues in proteins, a process known as N‐homocysteinylation (Sharma et al. 2015). This post‐translational modification alters protein folding and charge distribution, leading to loss of function, aggregation, and enhanced recognition as neoantigens, thereby initiating inflammatory and proteotoxic cascades.

Recent chemical studies have revealed additional reactivity of Hcy‐thiolactone. Loubane et al. (2023) demonstrated that its interaction with dehydroascorbic acid (DHA) forms covalent adducts that potentiate N‐homocysteinylation of peptides and proteins. These DHA–Hcy‐thiolactone conjugates generate novel carbonyl and sulfur linkages, promoting oxidative imbalance and expanding the spectrum of protein targets susceptible to homocysteinylation.

Beyond its biochemical reactivity, Hcy‐thiolactone exerts potent in vivo effects on neurovascular function. Yin et al. (2022) showed that Hcy‐thiolactone induces nitrosative stress and cognitive impairment through S‐nitrosylation of GTP cyclohydrolase 1 (GCH1), an enzyme essential for tetrahydrobiopterin (BH4) biosynthesis. This modification disrupts nitric oxide (NO) production, leading to endothelial dysfunction and impaired neurovascular coupling—hallmarks of vascular cognitive impairment.

At the neuronal level, N‐homocysteinylation has been implicated in protein aggregation and neurodegenerative processes. Masoumian Hosseini et al. (2025) reported that modification of lysine residues in α‐synuclein by Hcy‐thiolactone increases its aggregation propensity and cytotoxicity in SH‐SY5Y neuroblastoma cells, offering a direct biochemical connection between HHcy and synucleinopathies such as Parkinson's disease. In line with this, deficiencies in Hcy‐thiolactone–detoxifying enzymes, including paraoxonase 1 (PON1) and bleomycin hydrolase (BLMH), have been linked to Alzheimer's disease pathology (Jakubowski 2024), suggesting that inadequate clearance of Hcy‐thiolactone contributes to neurodegenerative risk.

Promisingly, therapeutic modulation of N‐homocysteinylation has been explored as a potential intervention strategy. Bhattacharya et al. (2025) demonstrated that proline competitively inhibits the homocysteinylation of lysine residues by binding to Hcy‐thiolactone, thereby attenuating protein damage and cytotoxicity. Such findings highlight a new biochemical approach for mitigating the molecular consequences of elevated homocysteine levels.

Taken together, these findings position Hcy‐thiolactone as a central effector of HHcy toxicity. Far from being a passive byproduct, it acts as a reactive metabolite that amplifies oxidative, nitrosative, and proteostatic stress, ultimately compromising neuronal and vascular homeostasis. Elucidating its reaction mechanisms, structural intermediates, and detoxification pathways may reveal novel therapeutic targets for preventing neurovascular deterioration in hyperhomocysteinemic conditions.

## 5 HHcy and Memory

**FIGURE 3:**
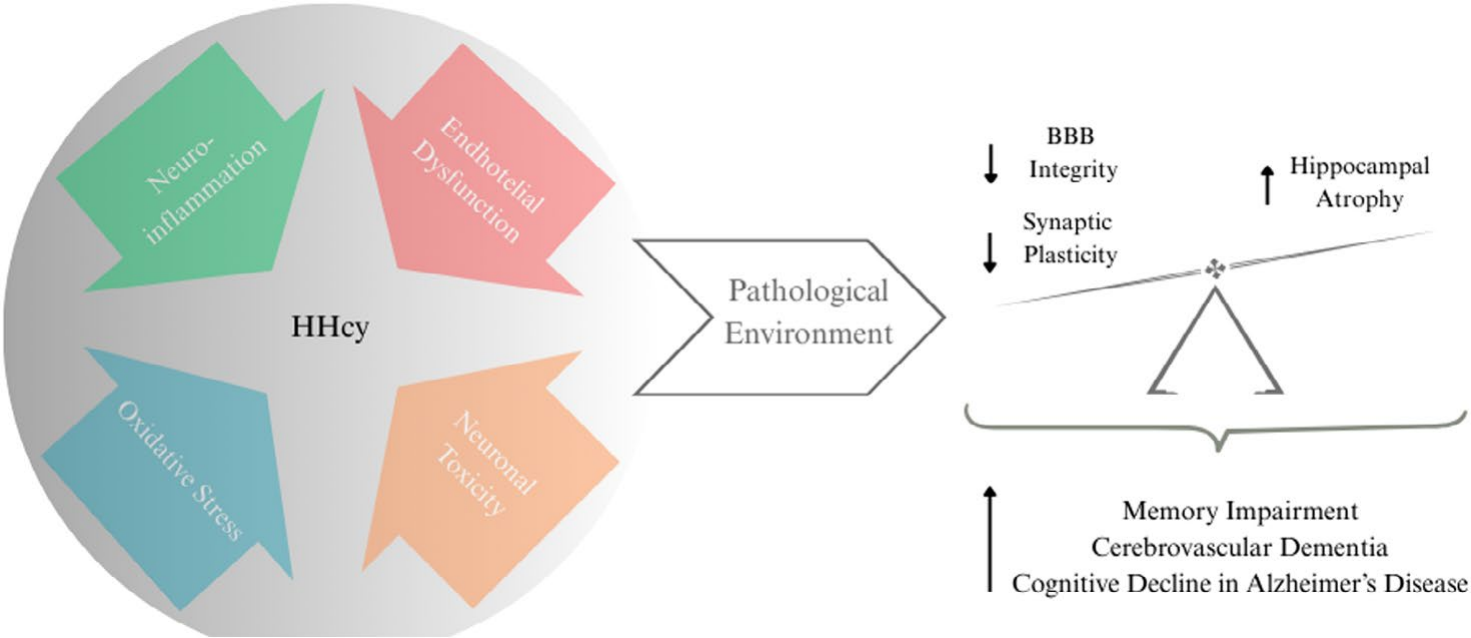
Hyperhomocysteinemia (HHcy) induces a pathological environment that disrupts the blood–brain barrier, reduces synaptic plasticity, and triggers neuronal degeneration in hippocampal regions. These alterations lead to memory impairment, exacerbate cerebrovascular dementia, and accelerate cognitive decline in AD.

As reported above, HHcy has been identified as a potential risk factor for neurodegenerative diseases that negatively impact memory, contributing to neurodegeneration through additional mechanisms such as oxidative stress, endothelial dysfunction, or neuronal toxicity (Miwa et al. 2015). This pathological environmental shift damages neurons and impairs synaptic plasticity, which is essential for memory formation (Figure 3).

Liu and colleagues demonstrated that HHcy‐induced memory impairment in rats was associated with neuronal lesions and degeneration in the hippocampal CA3 region—a critical area for memory consolidation—along with reduced synaptic density and downregulation of CREB phosphorylation, a key factor for synaptic plasticity and memory formation. Behavioral analyses revealed these animals exhibited significant deficits in spatial learning (Morris Water Maze), contextual memory (fear conditioning) (Liu, Zhao, et al. 2022). Further supporting these findings, studies showed that Hcy treatment disrupts essential hippocampal neuroplasticity pathways, including brain‐derived neurotrophic factor (BDNF)/tropomyosin receptor kinase B (TrkB) and ERK/mitogen‐activated protein kinase (MAPK) signaling (Algaidi et al. 2006; Tchantchou et al. 2023; Wei et al. 2014). Additionally, gestational HHcy exposure impaired long‐term potentiation (LTP) in the CA3 and CA1 regions of offspring at both 20 days (young) and 90 days (adults), in an NMDA receptor‐dependent manner. This was associated with delayed hippocampal maturation and dendritic spine development, ultimately leading to deficits in object recognition and spatial memory (Postnikova et al. 2022). At hippocampal CA1 and dentate gyrus subregions, Wyse and colleagues found that mild chronic HHcy altered biochemical and histological parameters, induced atrophy and impaired short‐term aversive memory (Wyse et al. 2020). Additionally, Ramires and colleagues showed that mild HHcy treatment affected spatial working memory, presynaptic hippocampal protein content of synapsin 1—a marker of synaptic plasticity—hippocampal neuroinflammatory process and motor coordination of rats (Ramires Junior et al. 2022).

Another factor induced by HHcy that compromises memory is vascular dementia; this condition accelerates cerebral small vessel disease, causing white matter hyperintensities, microbleeds, and lacunar infarcts. It disrupts the BBB and increases amyloid‐β deposition and microtubule‐associated protein tau (TAU) pathology, even in AD (Hainsworth et al. 2016). Zuliani et al. (2024) demonstrated that elevated plasma homocysteine (Hcy) levels are a significant predictor of progression from mild cognitive impairment (MCI) to dementia. This finding aligns with earlier work by Hooshmand et al. (2013), who analyzed post‐mortem brains of individuals aged 85+ years and found that high Hcy levels were associated with both Alzheimer's disease (AD) and cerebral vascular dementia (Hooshmand et al. 2013; Zuliani et al. 2024). Additional studies using AD‐compatible transgenic mice with HHcy demonstrated increased amyloid‐β and TAU pathology, synaptic dysfunction, and neuroinflammation. At the molecular level, the 5‐lipoxygenase, widely distributed in the CNS, plays a central role in mediating these HHcy‐induced effects. The upregulation and activation of the 5‐lipoxygenase pathway are necessary for Hcy‐dependent pathological effects in AD development (di Meco et al. 2018). Such findings suggest that HHcy may similarly worsen cognitive performance, particularly in domains like memory, executive function, and information processing speed, while potentially accelerating the rate of cognitive decline (Li et al. 2017; Luzzi et al. 2021).

Another factor is that HHcy has been strongly linked to insulin resistance and metabolic dysfunction, as demonstrated in a CBS heterozygous mouse model exhibiting elevated HHcy, glucose intolerance, and impaired insulin signaling. These effects were driven by increased hepatic and adipose tissue ROS production, mitochondrial dysfunction, and altered expression of genes regulating glucose homeostasis (Cruciani‐Guglielmacci et al. 2022). In humans, HHcy exacerbates metabolic disturbances, particularly in type 2 diabetes (T2D), where impaired renal clearance of homocysteine and disrupted folate/B12 metabolism further aggravate insulin resistance, β‐cell dysfunction, and microvascular complications (Mursleen and Riaz 2017). Notably, clinical studies reveal that T2D patients with mild cognitive impairment (MCI) show significantly higher Hcy levels, which correlate with deficits in attention, cognitive flexibility, and decision‐making, positioning HHcy as a promising biomarker for early cognitive decline in this population and highlighting its potential role in clinical risk stratification (Tian, Han, et al. 2017).

Growing evidence demonstrates a strong bidirectional relationship between Alzheimer's disease (AD) and metabolic dysfunction, with studies showing that AD patients exhibit cerebral insulin resistance that impairs glucose uptake necessary for synaptic function while simultaneously promoting amyloid‐β accumulation and tau hyperphosphorylation through activation of kinases like GSK‐3β (see Peng et al. 2024). This pathological overlap is further highlighted by epidemiological data showing type 2 diabetes (T2D) patients have a 50%–65% increased risk of developing AD (Rojas et al. 2021). At the molecular level, these conditions share common features such as chronic inflammation and mitochondrial dysfunction, leading some researchers to propose “Type 3 Diabetes” as a classification for AD cases with prominent insulin signaling disruption (la Monte and Wands 2008; Kciuk et al. 2024). These findings underscore the importance of glycemic control in at‐risk populations as a potential strategy to mitigate AD development, with emerging evidence suggesting that early metabolic interventions may preserve cognitive function by maintaining proper insulin signaling in vulnerable brain regions (Nguyen et al. 2020).

## 6 Conclusion

The present review provides a comprehensive examination of the complex regulatory factors of Hcy in clinical conditions. The interplay between dietary patterns, aging, hormonal influences, genetic mutations, medications, and interventions creates an intricate web that affects both the CNS and the cardiovascular system. Understanding these relationships is crucial, highlighting the impact of diets, the importance of age as an aggravating factor, the distinct roles of gender and hormones, the consequences of pharmacological interventions, and the relevance of genetic mutations. Furthermore, exploring the behavioral and molecular implications of Hcy in neurological and cardiovascular disorders adds a layer of understanding to these mechanisms. Given this panorama, the search for personalized intervention strategies is essential, considering the specific factors of each individual to optimize clinical management and prevent complications associated with Hcy. This in‐depth examination highlights gaps in existing research, suggesting promising areas for future investigation and emphasizing the importance of a holistic approach to fully elucidate Hcy regulation in clinical settings.

## Author Contributions

**Osmar Vieira Ramires Júnior:** conceptualization, investigation, writing – original draft, writing – review and editing, formal analysis, supervision, methodology, visualization. **Gustavo Ricardo Krupp Prauchner:** conceptualization, investigation, writing – original draft, writing – review and editing, methodology, formal analysis. **Alessandra Schmitt Rieder:** conceptualization, investigation, writing – original draft, writing – review and editing, methodology, formal analysis. **Ana Karla Oliveira Leite:** conceptualization, investigation, writing – original draft, methodology, writing – review and editing, formal analysis. **Clarissa Penha Farias:** conceptualization, investigation, writing – original draft, writing – review and editing, methodology, formal analysis. **Angela T. S. Wyse:** conceptualization, investigation, funding acquisition, writing – review and editing, methodology, formal analysis, supervision, resources.

## Funding

This study was supported by Edital Universal (405128/2021‐5)/Conselho Nacional de Desenvolvimento Científico e Tecnológico (CNPq), Instituto Nacional Saúde Cerebral (INSC, No 406020/2022‐1/CNPq).

## Ethics Statement

The authors have nothing to report.

## Consent

The authors have nothing to report.

## Conflicts of Interest

The authors declare no conflicts of interest.

## Data Availability

The data that support the findings of this study are available from the corresponding author upon reasonable request.
